# Flux-sum analysis: a metabolite-centric approach for understanding the metabolic network

**DOI:** 10.1186/1752-0509-3-117

**Published:** 2009-12-19

**Authors:** Bevan Kai Sheng Chung, Dong-Yup Lee

**Affiliations:** 1 NUS Graduate School for Integrative Sciences and Engineering, National University of Singapore, 28 Medical Drive, #05-01, 117456, Singapore; 2 Bioprocessing Technology Institute, Agency for Science, Technology and Research (A*STAR), 20 Biopolis Way, #06-01 Centros, 138668, Singapore; 3 Department of Chemical and Biomolecular Engineering, National University of Singapore, 4 Engineering Drive 4, 117576, Singapore

## Abstract

**Background:**

Constraint-based flux analysis of metabolic network model quantifies the reaction flux distribution to characterize the state of cellular metabolism. However, metabolites are key players in the metabolic network and the current reaction-centric approach may not account for the effect of metabolite perturbation on the cellular physiology due to the inherent limitation in model formulation. Thus, it would be practical to incorporate the metabolite states into the model for the analysis of the network.

**Results:**

Presented herein is a metabolite-centric approach of analyzing the metabolic network by including the turnover rate of metabolite, known as flux-sum, as key descriptive variable within the model formulation. By doing so, the effect of varying metabolite flux-sum on physiological change can be simulated by resorting to mixed integer linear programming. From the results, we could classify various metabolite types based on the flux-sum profile. Using the *i*AF1260 *in silico *metabolic model of *Escherichia coli*, we demonstrated that this novel concept complements the conventional reaction-centric analysis.

**Conclusions:**

Metabolite flux-sum analysis elucidates the roles of metabolites in the network. In addition, this metabolite perturbation analysis identifies the key metabolites, implicating practical application which is achievable through metabolite flux-sum manipulation in the areas of biotechnology and biomedical research.

## Background

Cellular metabolism is more often than not represented and analysed based on a stoichiometric modelling framework under the stationary assumption of the metabolic network [1]. Such stationary approaches, e.g. flux balance analysis (FBA), circumvent issues related to kinetic modeling, including the lack of experimental data and the need for estimation of kinetic parameters, and provide useful information about the characteristics of the system as evident in various nonlinear dynamic analysis techniques [2]. Furthermore, the assumption of metabolic steady-state is usually valid since the intracellular dynamics are typically much faster than extracellular dynamics [1] and metabolite concentrations generally equilibrate in a much shorter time (in seconds) compared to the time-scale of genetic regulation (in minutes) [3-5]. Consequently, the constraint-based reconstruction and analysis (COBRA) approach provides an elegant method of characterizing and predicting cellular phenotype and metabolic states through the application of FBA which solves a linear optimization problem by assuming some form of cellular objective as the performance criterion [6-8].

Metabolites and biochemical reactions in the metabolic network can be graphically represented by nodes and edges connecting the nodes respectively. Based on this graphical representation, it is obvious that there can be two approaches in the analysis of the network, focusing on the flow of materials through either the nodes (metabolite-centric approach) or edges (reaction-centric approach). Typical FBA can be conducted based on a reaction-centric approach where constraints were introduced to restrict the range of reaction flux values so as to define a feasible solution space [9]. This analysis is intrinsically reaction-centric since reaction fluxes are the key description variables in the model formulation [10]. Previous studies involving the application of FBA mostly dealt with gene or reaction knockouts [11-13] and manipulation of reaction rates [14], which examined the phenotypic morphology resulting from the alteration of reaction fluxes. These reaction-centric approaches, especially in [14], provided us with a quantitative understanding of the reaction essentiality in a metabolic network.

On the other hand, the metabolite-centric approach towards addressing metabolite essentiality was, to date, only attempted by a handful of studies [15-17] which mostly presented qualitative effects of removing metabolites from the network. Only [16] demonstrated the use of a quantitative measure of metabolite essentiality known as the "flux-sum" which indicates the turnover rate of a particular metabolite. Recognizing the fact that metabolites play important roles in shaping the metabolic network [18], we propose a methodology for the quantitative analysis of metabolite essentiality that can overcome the limitations of the previous formulation and extend the scope of analysis. As the constraint-based modeling, we incorporated metabolite flux-sum constraints with the reaction flux constraints in the mathematical formulation so as to investigate the effect of varying metabolite flux-sum on cellular metabolism and phenotypic change. The efficacy and usefulness of this metabolite-centric approach was demonstrated by applying it to the *E. coli *system using *i *AF1260 *in silico *metabolic model [19].

## Methodology

### Defining the flux-sum

The constraint-based analysis of the metabolic network evaluates the steady-state flux distribution which satisfies the flux balance condition:  for any internal/intermediate metabolites *i*, where *S*_*ij *_refers to the stoichiometric coefficient of metabolite *i *participating in reaction *j *and *v*_*j*_, the flux of reaction *j*. At pseudo-steady-state, there is no accumulation of intermediate metabolites but the absolute rate of metabolite consumption or production can be nonzero. Therefore, we can define a new descriptive variable of "flux-sum" to represent the turnover rate of a metabolite by summing up all the incoming or outgoing fluxes around the metabolite [16]. This definition clearly indicates that the unit of flux-sum is equivalent to that of the reaction flux (i.e. mmol/g-DCW-hr). Hence, we let Φ_*i *_denote the flux-sum of metabolite *i *and its mathematical form is given by . This variable can be further constrained to explore phenotypic changes under perturbed conditions such as attenuation or intensification in the metabolic network.

### Flux-sum constraint with binary variables

The nonlinear flux-sum term within the mathematical formulation was originally recast into a series of linear constraints based on the mathematical relationship (*a *- *b*)^2 ^≤ (*a *+ *b*)^2 ^which leads to |*a *- *b*| ≤ *a *+ *b *only under the condition that *a *≥ 0 and *b *≥ 0 [16,20]. By introducing two positive variables *a*_*j *_and *b*_*j*_, they let *S*_*ij*_*v*_*j *_= *a*_*j *_- *b*_*j *_for metabolite *i*. Thus, the constraint  effectively ensured that the flux-sum of metabolite *i*, Φ_*i*_, is less than or equal to the value of *C*. Since this method enables us to specify "≤" constraints on flux-sums, it sufficed the analysis of flux-sum attenuation. However, this technique is inadequate for the implementation of flux-sum intensification, which requires "≥" constraints. In order to overcome the limitation, we reformulate flux-sum constraints as follows: Similarly, we let the rate of consumption/production of metabolite *i *due to reaction *j *be expressed in terms of two positive variables: , where  and . Thus the flux-sum of metabolite *i *can be expressed as . We observed that  if and only if either  or , or simply . Then, the flux-sum of metabolite *i *is given by  when either  or . This condition can be satisfied by introducing indicators or binary variables:  and , and the constraints ; ; and . Note that big *M *is some finitely large number that should at least be larger than the largest possible reaction flux observed experimentally. The rationale for using big *M *instead of infinity is that the product of zero and infinity is undefined. This formulation of flux-sum circumvents the "bad" nonlinear constraint [20].

### Flux-sum analysis

In order to conduct flux-sum attenuation and intensification analyses, we need reference values representing the *base case *and the range of feasible flux-sum values. The collection of flux-sum values corresponding to the unperturbed system or base case would be referred to as the *basal *flux-sum distribution that can be calculated from the flux distribution as a result of FBA. The minimum flux-sum value of any metabolite is set as zero since Φ_*i *_≥ 0 while the maximum flux-sum can be evaluated using the new mathematical formulation that is elaborated below. Based on these reference values, we can attenuate or intensify each metabolite's flux-sum by gradually decreasing or increasing the flux-sum value from the basal value to zero or the maximum value, respectively. In summary, the entire process of flux-sum analysis is carried out in 3 steps:

**Step 1**: Evaluate basal flux-sum distribution.

**Step 2**: Evaluate flux-sum maxima of individual metabolites.

**Step 3**: Manipulate flux-sum by attenuation and intensification.

The mathematical details for every step of the procedure are described as follows:

#### Step 1: Evaluate basal flux-sum distribution

The basal flux-sum distribution can be evaluated based on the "wild-type" flux distribution which is determined by solving the following FBA formulation under the unperturbed or normal condition:(P1)

In this study, we assumed the cellular objective to be biomass formation (or cell growth), *v*_*biomass*_. The preceding formulation allowed the input of experimental observations by specifying the values of *α*_*j*_, *β*_*j*_, *λ*_*i *_and *μ*_*i*_. In the case where upper and lower bounds of fluxes are unavailable, the flux capacities can be set as *α*_*j *_= 0, *β*_*j *_= +inf for irreversible reactions and *α*_*j *_= -inf, *β*_*j *_= +inf for reversible reactions. Similarly, for metabolite uptake or secretion constraints on exchange fluxes (*v*_*EX_i*_), we can set *λ*_*i *_= 0, *μ*_*i *_= +inf for metabolites that are secreted only; *λ*_*i *_= -inf, *μ*_*i *_= 0 for metabolites that are consumed only; and *λ*_*i *_= -inf, *μ*_*i *_= +inf for metabolites that can enter and leave the system freely.

After solving **(P1)**, the basal flux-sum value of any metabolite *i *is calculated using the formula , indicating the summation of all incoming or outgoing fluxes around metabolite *i *under the normal condition.

#### Step 2: Evaluate flux-sum maxima of individual metabolites

The flux-sum maxima of metabolites can be calculated by solving the following mixed-integer optimization (MIP) problem:(P2)

We let  denote the maximum flux-sum value of **(P2) **for metabolite *i*.

#### Step 3: Manipulate flux-sum by attenuation or intensification

With the reformulation of flux-sum constraints we fix the flux-sum of any metabolite at a particular value and evaluate the corresponding metabolic state. In order to ensure feasibility, the basal flux-sum can be considered as the starting point, followed by examining the effects of decreasing and increasing metabolite flux-sums through flux-sum attenuation and intensification analysis, respectively. The mathematical formulation for this analysis is given as follow:(P3)

By solving this MIP problem, we can obtain the biomass production values for different levels of flux-sum attenuation or intensification. Note that either **(C1) **or **(C2) **is implemented depending on whether the problem is flux-sum attenuation or intensification respectively. The parameters *k*_att _and *k*_int _control the levels of flux-sum attenuation and intensification respectively. Initially, setting *k*_att _= 1 or *k*_int _= 0 constrains the flux-sum at the basal level. Subsequently, we can attenuate or intensify the flux-sum by decreasing *k*_att _or increasing *k*_int _until *k*_att _= 0 or *k*_int _= 1, where the flux-sum would reach zero or the maximum value, respectively. The decrement and increment of *k*_att _and *k*_int _can be in steps of 0.1 so that they only take on values from the set {0, 0.1, 0.2 ... 1.0}.

## Application

### *In silico *model settings

The genome-scale *in silico E. coli *model *i*AF1260 was employed to demonstrate the efficacy and applicability of the current flux-sum approach. The model was made up of 1668 metabolites (951 cytoplasmic and 418 periplasmic intermediates and 299 external metabolites) and 2382 reactions including the biomass reaction, thus making up a 1668 by 2382 stoichiometric matrix [18]. In the current model, the metabolites are compartmentalized. Hence, we distinguish same metabolites in different compartments using suffixes [c], [p] and [e] for cytosol, periplasm and extracellular matrix respectively. For example H_2_O [c], H_2_O [p] and H_2_O [e] indicate water found in three different compartments. The cellular objective was assumed to be the maximization of biomass production. The reaction reversibility constraints were set as given by [19]. The non-growth associated maintenance energy is maintained at 8.39 mmol ATP/(g-DCW-hr) while the maximum glucose and oxygen uptake rates were assumed as 10 mmol/(g-DCW-hr) and 20 mmol/(g-DCW-hr) respectively to simulate the aerobic growth condition of *Escherichia coli *in glucose minimal medium. All these settings were based on previous observation for glucose and oxygen uptake rates and experimentally determined ATP requirement for maintenance [6,19,21]. The GAMS IDE software version 22.4 was used to solve all the mathematical programming problems in this study.

### Basal flux-sum

**Figure 1:**
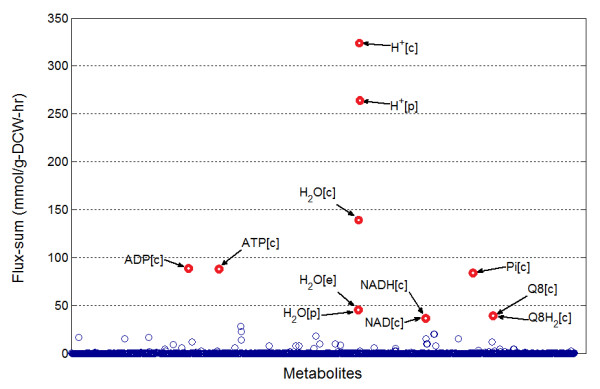
**Basal flux-sum distribution**. Metabolites with ultra-high basal flux-sum values were cofactors like ATP, ADP and H^+^. It was also observed that a large number of metabolites (i.e. 795 or 58.1%) were not utilized and many of them were found to be *blocked *metabolites.

**Figure 2:**
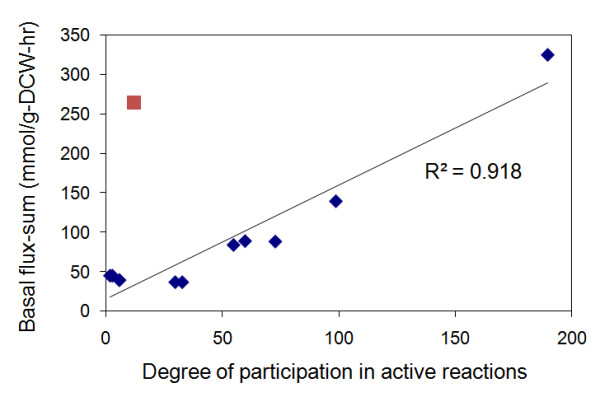
**Basal flux-sum vs degree of participation**. Most of the 17 metabolic cofactors with high flux-sum were highly connected and participated in proportionally as many basal active reactions, except for periplasmic hydrogen ions (red marker). The R-square value for the linear relationship was 0.912 without considering the outlier (red marker). The 17 metabolites are ADP [c], ATP [c], CO_2 _[c], H_2_O [c], H^+ ^[c], NAD [c], NADH [c], PI [c], Q_8 _[c], Q_8_H_2 _[c], CO_2 _[p], H_2_O [p], H^+ ^[p], O_2 _[p], CO_2 _[e], H_2_O [e] and O_2 _[e]. Only ADP [c], ATP [c], H_2_O [c], H^+ ^[c], H^+ ^[p] and PI [c] have ultra-high flux-sum.

We generated a basal metabolite flux-sum distribution (Figure 1) for the *i*AF1260 model by solving **(P1)**, resulting in 4.20 and 23.9 for average (*μ*_*FS*_) and standard deviation (*sd*) of the flux-sum values respectively. About 70% of the metabolites have zero flux-sum in the base case while metabolites with high () and ultra-high ((1)) basal flux-sums were mostly essential cofactors that are involved in oxidative phosphorylation and redox reactions with a high degree of participation in basal active reactions (i.e. reactions with nonzero basal flux). We define the degree of participation of a metabolite as the number of instances the metabolite is involved in an active reaction. Figure 2 indicates a linear relationship between the flux-sum values and the degree of high flux-sum metabolites which were previously identified as giant strong components forming critical links in the metabolic network [22]. The fluxes of oxidative phosphorylation reactions contributed to at least 50% of the flux-sum of these metabolites. Among them, periplasmic hydrogen ion appeared to be an outlier due to the low degree of participation in metabolic reactions other than oxidative phosphorylation. Such a low degree of participation of periplasmic hydrogen ion is mainly due to the scarcity of periplasmic metabolic reactions.

### Flux-sum maxima

**Figure 3:**
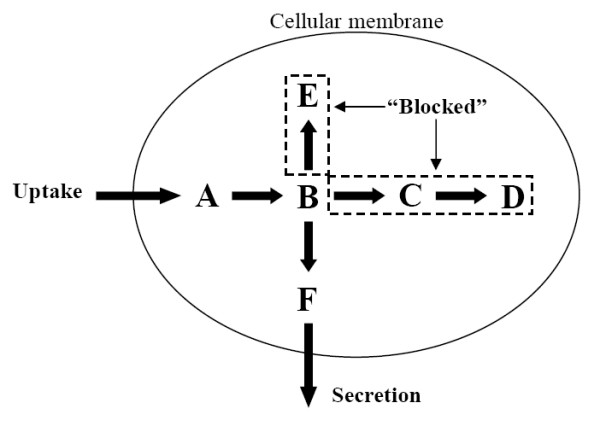
**Dead-ends and blocked metabolites**. In a simple system involving metabolites A, B, C, D, E and F, metabolites D and E are pathway dead-ends. Reactions B → C, B → E and C → D are blocked reactions since metabolites D and E are neither consumed nor secreted. Consequently, metabolites C, D and E are blocked metabolites.

Interestingly, the evaluation of flux-sum maxima, obtained from solving **(P2)**, allowed us to identify different types of metabolites. Firstly, we found *blocked *metabolites with maximum flux-sum equal to zero. The consumption and production of these metabolites were blocked due to reaction pathway *dead-ends *(Figure 3). The *blocked *metabolite is analogous to the *blocked *reaction reported by [23]. Thus, similarly we can define *unconditionally blocked *metabolites as metabolites with zero maximum flux-sum even when all of the exchange fluxes were completely unconstrained. Removing all the reactions associated with unconditionally blocked metabolites can reduce the size of the stoichiometric matrix without affecting the simulation results. For the *i*AF1260 model, it was observed that 442 and 189 intermediate metabolites were conditionally and unconditionally blocked, respectively, under the aerobic glucose minimal medium condition.

Secondly, we identified 75 *cyclic *metabolites involved in internal cycles, also known as Type III pathways [24], in the *i*AF1260 model. *Cyclic *metabolites have maximum flux-sums equal to infinity regardless of any substrate uptake constraint imposed on the system since any rate of production of such metabolites can be balanced by the same rate of consumption within the cycle. Therefore, the determination of flux-sum maxima provides an alternative method for identifying Type III pathways.

Lastly, we identified 55 *fully utilized *metabolites with nonzero maximum flux-sum which are equal to their basal flux-sum values. As the cell strives for maximal growth, these metabolites are turned over at their full capacity. On the other hand, 797 *partially utilized *metabolites are not turned over at their full capacity during maximum cell growth, thus their flux-sums can be further intensified. This phenomenon is further examined in a later section. Note that some *partially utilized *metabolites may be basal inactive.

### Flux-sum attenuation analysis

**Figure 4:**
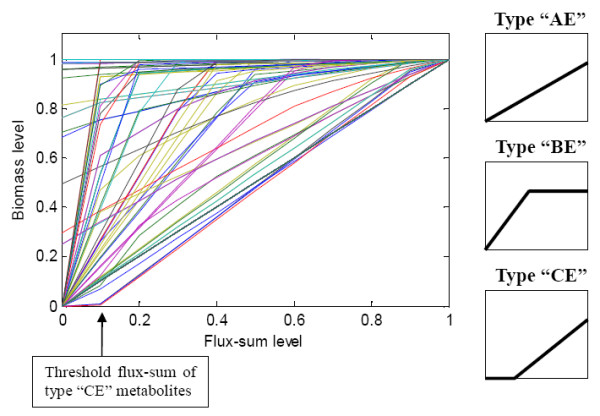
**Flux-sum attenuation profile**. The horizontal axis corresponds to the value of *k*_att_. The vertical axis corresponds to the biomass production normalized with respect to the basal biomass production. Profiles of essential metabolites intersect the origin and each could be classified as either type "AE", "BE" or "CE", with the suffix "E" indicating that the metabolite is essential.

In the *i*AF1260 model, 394 out of 1369 intermediate metabolites had a nonzero basal flux-sum and were amenable for flux-sum attenuation analysis. Thus, we solved **(P3) **with constraint **(C1) **for these 394 basal active metabolites, thereby giving rise to the flux-sum attenuation profile (Figure 4). From the profile, we identified 342 essential and 52 nonessential metabolites as those with zero and nonzero biomass production, respectively, at full flux-sum attenuation. Of the essential metabolites, some were involved in amino acid biosynthesis: tetrahydrodipicolinate, L,L-2,6-diaminopimelate and meso-2,6-diaminopimelate, and these metabolites were in fact associated with the essential genes reported by [25]. Thus, essential metabolites can be associated with lethal reactions and the removal of any of such metabolites or reactions leads to no cell growth. Hence, they signify critical points of fragility in the metabolic network. Interestingly, 86.8% of the basal active metabolites were essential metabolites while only 68.5% of the basal active reactions were lethal. The observed higher level of reaction redundancy would be attributable to the presence of redundant pathways that connect essential metabolites. This also elucidated that a metabolite associated with a lethal reaction would inevitably be essential while a reaction involving essential metabolite(s) might not necessarily be lethal.

The flux-sum attenuation profile (Figure 4) reproduced the general profiles of type "A", "B" and "C" essential metabolites as reported by [16]. In this study, we labeled the metabolites as type "AE", "BE" and "CE" with the suffix "E" indicating that the metabolites were essential. It is not surprising to observe type "AE" profile (304 out of 342 essential metabolites) since all constraints are linear and biomass production is expected to vary linearly with the synthesis of some metabolites. Interestingly, the type "CE" profile (6 out of 342 essential metabolites) showed a more rapid drop than type "AE" when the flux-sum was attenuated and these metabolites were found to be involved in providing the ATP requirement for non-growth associated maintenance (NGAM). The flux-sum threshold, below which biomass production is impossible, corresponds to the ATP requirement for NGAM. The amount of flux-sum in addition to this threshold value is then associated with biomass production. Thus, the threshold flux-sums of type "CE" metabolites allow us to calculate the distribution of the resources between growth and NGAM requirements. The peculiar shape of type "BE" profile (32 out of 342 essential metabolites) was attributed to the existence of alternate optimal solutions [26] where a small reduction of flux-sum can be compensated by other "equivalent" fluxes. When the flux-sums of these metabolites were further attenuated, there would be no "equivalent" compensation for synthesizing the essential biomass components. Thus the biomass production rate would drop below the optimal value and hit zero eventually.

**Figure 5:**
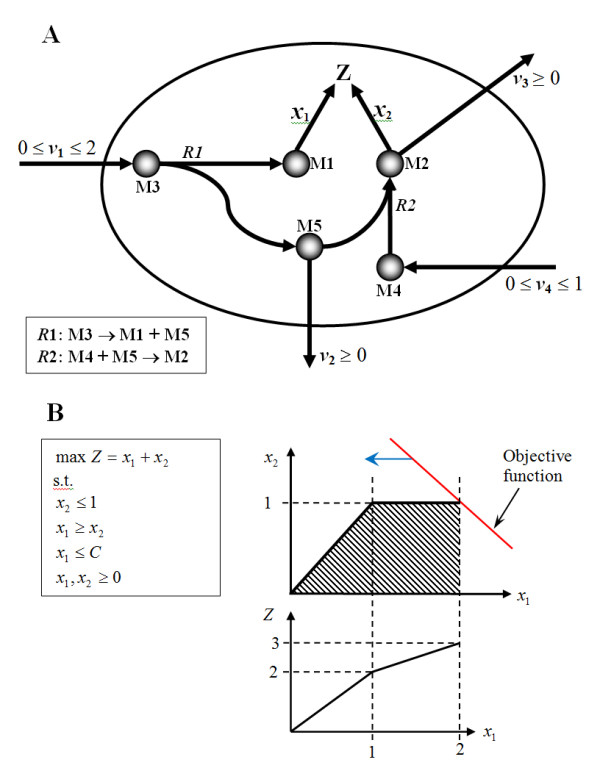
**A sample network containing a hybrid metabolite**. The cellular objective of the sample network (A) was to maximize *Z *and the system could be formulated as a linear programming problem (B). We attenuate the flux-sum of metabolite M1, which is also equal to *x*_1_, and examine the effects on the objective *Z*. When *x*_1 _was attenuated by decreasing the value of *C*, the maximum value of *Z *decreased as the objective function (red line) traversed the edges of the solution space (shaded region) in the direction shown by the blue arrow. As the objective function passes *x*_1 _= 1, the "rate" of decrease of *Z *changes due to the difference in gradients of the edges.

It was also observed that some metabolites exhibited a profile that seemed to be a hybrid between type "AE" and "BE". This can be due to the traversing of the optimal solution across linear edges of the solution space with different gradients as the attenuation of the flux-sum reduced the solution space. These gradients of the edges in the solution space can be interpreted as the sensitivity of biomass production to the alteration of flux-sum. A simple reaction network is used to illustrate how the hybrid profile is generated (Figure 5).

### Flux-sum intensification analysis

In flux-sum intensification analysis, we examined how the increase of metabolite flux-sum affects the cell growth. 442 blocked, 75 cyclic and 55 fully utilized metabolites were omitted for this analysis due to infeasibility. Thus the flux-sum intensification analysis was only carried out for the remaining 797 *partially utilized *metabolites.

**Figure 6:**
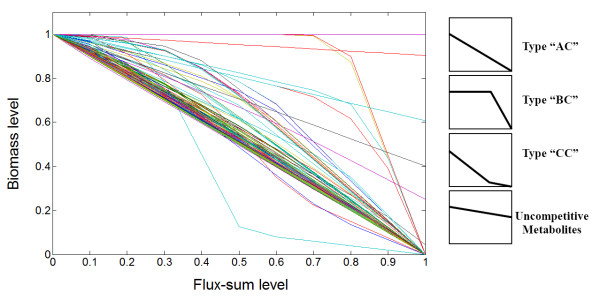
**Flux-sum intensification profile**. The horizontal axis corresponds to the value of *k*_int_. The vertical axis corresponds to the biomass production normalized with respect to the basal biomass production. Metabolites with profile that intersected the point (1, 0) were competitive metabolites which could be classified as either "AC", "BC" or "CC" with the suffix "C" indicating that it is competitive and the other metabolites are considered uncompetitive.

**Figure 7:**
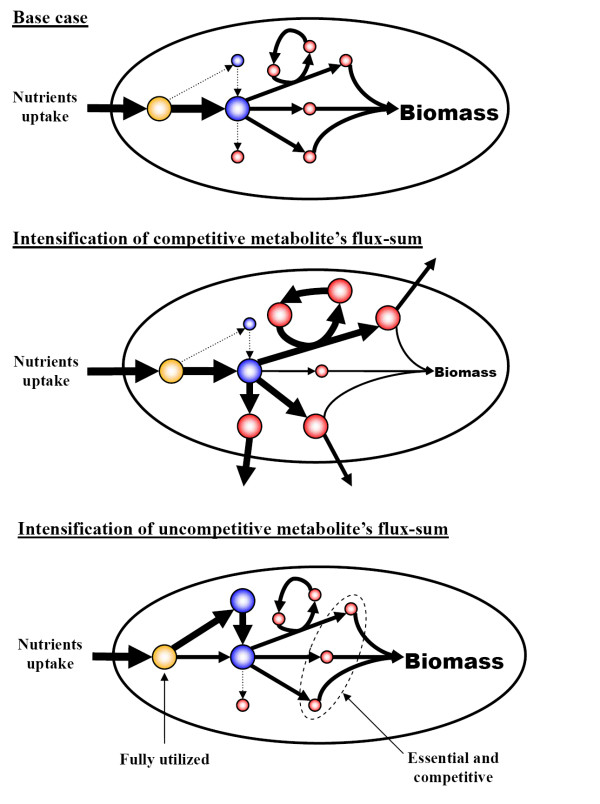
**Flux-sum intensification of competitive and uncompetitive metabolites**. In the base case, the maximum biomass production can be achieved due to optimal distribution of carbon fluxes to all the biomass components. If we intensify the flux-sum of any of the competitive metabolites (red nodes), the metabolite would compete for the limited resources and perturb the optimal distribution of carbon fluxes, resulting in reduced biomass production. On the other hand, intensifying the flux-sums of uncompetitive metabolites (blue nodes) does not perturb the optimal carbon flux distribution while the flux-sum of fully utilized metabolites (orange nodes) cannot be intensified. It is obvious that all metabolites contributing to biomass production shown in the figure are both essential and competitive.

By solving **(P3) **with constraint **(C2)**, we generated the flux-sum intensification profile (Figure 6). Then we classified the metabolites in a similar fashion as in flux-sum attenuation analysis. We defined *competitive *metabolites as the flux-sum intensification analogues for essential metabolites in the flux-sum attenuation case. Then, we classify competitive metabolites as type "AC", "BC" and "CC" based on the shape of the intensification profile (Figure 6), with the suffix "C" meaning competitive. Competitive metabolites compete for the same resources required for the biomass production. Thus, their complete flux-sum intensification resulted in zero cell growth. On the other hand, uncompetitive metabolites are probably cofactors in biomass production or some intermediates involved in alternate pathways for the production of biomass components, thus allowing the cell to grow even at 100% flux-sum intensification (Figure 7). In the *i*AF1260 model, we found 785 competitive metabolites and 12 uncompetitive metabolites.

## Discussion

### Flux-sum attenuation and intensification

**Figure 8:**
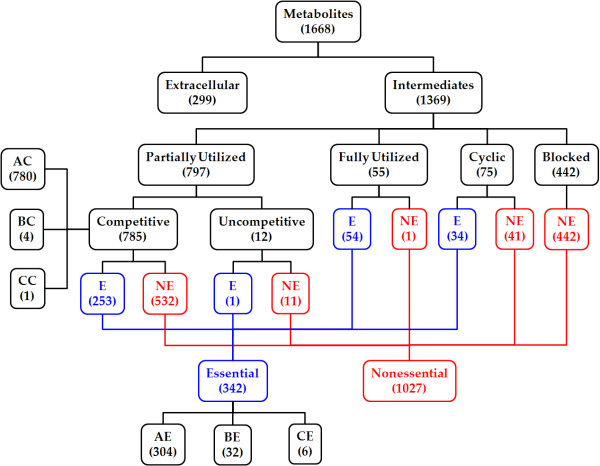
**Summary of metabolite classification**. The numbers of metabolites in each category is shown in brackets. The abbreviations "E" and "NE" denote essential and nonessential metabolites respectively. "AC","BC", "CC" and "AE", "BE", "CE" refer to the types of metabolites identified in flux-sum intensification and attenuation analyses respectively.

When the *E. coli *system was perturbed by attenuating and intensifying the metabolite flux-sums, we could observe various types of metabolites and classified them according to the profile shape (see Figure 8; refer to Additional file 1 for the full list of metabolites in each category). In order to understand the rationale of this classification, we further examined the biological relevance of such flux-sum attenuation and intensification. As the cell strives to maximize its growth, it fully utilizes its resources and distributes them optimally to synthesize the essential cellular components. This optimum distribution was determined by FBA of the unperturbed case. Based on the result, flux-sum attenuation of essential metabolites forces the cell to utilize less of its resources, leading to slower production of cellular components and subsequent slower growth. On the other hand, intensifying the flux-sum of competitive metabolites causes the suboptimal distribution of resources which also results in the slower cell growth. Thus intensifying the flux-sum of a competitive metabolite may attenuate the flux-sum of other essential metabolites.

**Figure 9:**
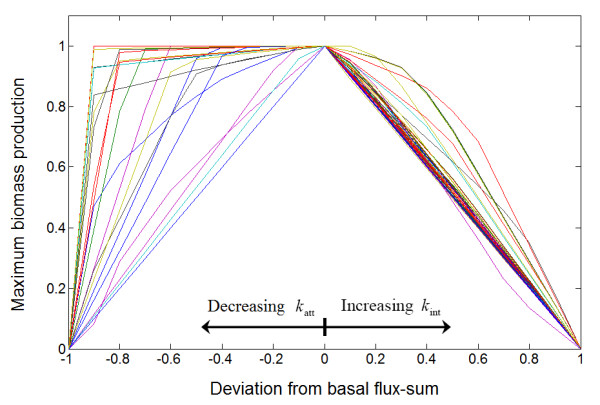
**Composite profile of essential and competitive metabolites**. This profile is considered a "composite" profile because different regions of *x*-axis represents different variables: negative values on the *x*-axis correspond to the value of (*k*_att _- 1) while the positive values correspond to the value of *k*_int_. When the *x*-axis is equal to zero, the flux-sum is at basal value where there is no attenuation or intensification.

From a network topological perspective, essential metabolites are found along the pathways synthesizing essential cellular components while competitive metabolites are from the pathways parallel to these essential ones, sharing at least one common metabolite precursor so that they can compete for the same resources. Competitive metabolites can also be essential if there are parallel essential pathways sharing common precursors such as 12 metabolites reported in [27]. The combination of flux-sum attenuation and intensification analyses allows us to identify 253 of such essential and competitive metabolites. Interestingly, these metabolites generally exhibited type "AC" profile during flux-sum intensification (Figure 9), indicating that the intensification of any essential metabolite in parallel pathways is very detrimental to the cell growth.

### Application of metabolite classification

The classification of metabolites based on flux-sum analysis, summarized in Figure 8, allows us to consider various practical applications. For example, in anti-pathogen study, researchers would be interested in developing strategies to identify targets which inhibit the growth of pathogens. In this sense, types "AE" and "CE" metabolites, identified through flux-sum analysis, serve as promising targets since the attenuation of their flux-sum may lead to the significant reduction in the cell growth. Similarly, types "AC" and "CC" metabolites can also be potential regulators affecting pathogenic growth through their flux-sum intensification. We also observed that fully utilized metabolites, except for periplasmic oxygen and cytosolic isopentenyl diphosphate, are fully coupled with the cell growth, thus indicating that these metabolites are perfectly correlated with the cell growth from the metabolite point of view. Note that the concept of flux coupling was discussed in [23]. Consequently, these metabolites can be considered as potential indicators for the cell viability as well as good metabolic engineering targets for controlling cell growth. As another application, the identification of blocked metabolites can be useful for improving the process of metabolic network reconstruction. During automated reconstruction, it is common to have gaps in the draft metabolic network which would result in the failure to predict experimentally observed cellular phenotypes and thus it is required to consider a systematic way to fill these gaps [28]. The first step in the gap-filling process is to identify the location of these gaps in the metabolic network. Through flux-sum analysis, we can identify metabolites which are *in vivo *essential and *in silico *blocked. Then appropriate metabolic reactions can be introduced into the incomplete metabolic network model, thus bridging the gap between these metabolites and the other *in silico *active metabolites.

### Biotechnological application of flux-sum analysis

In this study, we demonstrated the effects of changing metabolite flux-sums on the cell growth in *E. coli*. In a similar vein, we can also analyze the effects of metabolite flux-sums on the production of desired biomolecules for the biotechnological application. Here, we carried out flux-sum attenuation and intensification analyses, thereby identifying potential metabolite targets to be manipulated so as to increase anaerobic succinate production in *E. coli *(see Additional file 2). Surprisingly, we found that pyruvate is the only candidate for flux-sum attenuation leading to the enhanced production of succinate. This result was previously validated in an experiment whereby knocking out the genes of pyruvate producing and assimilating enzymes increased succinate production in *E. coli *[29]. In addition, we also identified flux-sum intensification targets, such as glyoxylate, 2-phosphoglycerate and 3-phosphoglycerate, which were also reported as effective targets for increasing succinate production (see Additional file 2).

## Conclusions

This paper presents a novel method for analyzing the metabolic network using a metabolite-centric approach within the context of constraint-based flux analysis. We utilized flux-sum constraints to understand the role of metabolites and apply this knowledge to generate testable hypotheses about the relationship between target metabolites and physiological changes, indicating the potential application of the metabolite-centric approach to biomedical research. Flux-sum analysis was also shown to be useful in the biotechnological application for improving the production of desired metabolite such as succinate in *E. coli*. In summary, the flux-sum analysis methodology can be considered as a useful technique providing better understanding of the cellular metabolism and alternative perspectives on how to engineer the system.

## Authors' contributions

BKSC and DL developed the modeling approach. BKSC did the computational simulations and drafted the manuscript. DL revised the manuscript. All authors read and approved the final manuscript.

## Supplementary Material

Additional file 1 **List of different types of metabolites identified in this study.** Metabolites are abbreviated in a similar fashion as in Feist *et al*. (2007). Click here for file

Additional file 2 Demonstration of flux-sum analysis for increasing succinate production in *Escherichia coli*. Click here for file
