# Supplementary material for: Flux-sum analysis: a metabolite-centric approach for understanding the metabolic network
Source: BMC Syst Biol. 2009 Dec 19;3:117. doi: 10.1186/1752-0509-3-117 (PMC2805632; doi:10.1186/1752-0509-3-117)
Supplement: Additional file 2 — Demonstration of flux-sum analysis for increasing succinate production in Escherichia coli. [file 1752-0509-3-117-S2.DOC]

# Supplementary information

## Flux-sum analysis for succinate production in *Escherichia coli*

A study on *Escherichia coli* strain improvement revealed that disrupting genes for pyruvate-forming enzymes leads to enhanced succinate production (Lee et al., 2005). In that study, different combinations of gene disruptions were simulated *in silico* and a gene knockout combination of *ptsG*, *pykF* and *pykA*, which increases the production of succinate by reducing the formation of pyruvate, was identified. In this case study, we demonstrate that the flux-sum attenuation analysis can arrive at the same results more effectively compared to previous methodologies.

By evaluating the anaerobic succinate production during flux-sum attenuation, we can identify a prominent profile for only one metabolite, which is pyruvate. Since we cannot be sure if any alternative optimum is present which can lead to lower succinate production, we carried out further *in silico* simulations to determine the minimum value of succinate production given a certain level of flux-sum attenuation and biomass production constraint. The mathematical formulation for this simulation, given by equation **P4** (refer to page 6), is similar to the original flux-sum attenuation analysis except for the additional biomass constraint and the different objective function of minimizing succinate production.


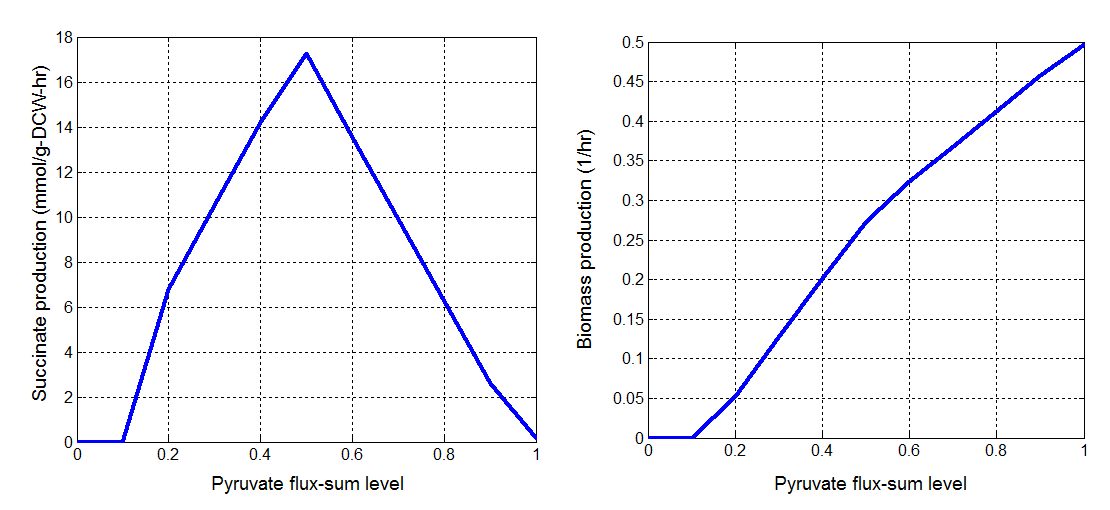


**Figure S1: Pyruvate flux-sum attenuation profile.** The horizontal axis shows the value of *k_att_* so the smaller the value, so the greater the degree of flux-sum attenuation. Succinate production values shown on the left are the minima found by solving **P4**.

By evaluating the minimum succinate production using **P4**, we found that the attenuation of pyruvate flux-sum forced *E. coli* to produce at least the minimum value of succinate production rate as the cell attempts to achieve its maximal biomass production (Figure S1). The attenuation of pyruvate flux-sum was experimentally demonstrated by Lee et al. (2005), through the disruption of pyruvate producing genes (*ptsG*, *pykA* and *pykF*), to be effective in increasing succinate production and further disruption of pyruvate-dissimilating genes, *ldh* and *pfl*, can inhibit the formation of other by-products but at the cost of slower cell growth. Moreover in this study, the flux-sum profile also indicated that there is an optimal level of flux-sum attenuation (i.e. at 50 %) below which succinate production would be reduced instead of increased and this could potentially serve as an interesting research problem where metabolic engineers would attempt to control the flux-sum of pyruvate at that particular optimal state.

In addition, flux-sum intensification analysis also revealed several metabolites which flux-sums are positively related to succinate production (Figure 2). The intensification of the flux-sums of these metabolites leads to increased succinate production but decreased biomass production. For example, increasing the flux-sum of glyoxylate, *cis*-aconitate or isocitrate can result in increased succinate production. Sanchez et al. (2005) had reported that increasing fluxes through the glyoxylate pathway by knocking out *adhE*, *ldhA*, *ack*-*pta* and *iclR* genes can enhance succinate production. Since glyoxylate, *cis*-aconitate and isocitrate are metabolites in the glyoxylate pathway, the experimental observation provided evidence for the relationship between the flux-sum of these metabolites and succinate production. Furthermore, Wang et al. (2006) had also shown that a *ptsG* and *iclR* double knockout *E. coli* strain was capable of high succinate production, indicating that the combination of pyruvate flux-sum attenuation (achieved by *ptsG* knockout) and glyoxylate pathway metabolites flux-sum intensification (achieved by *iclR* knockout) can be effective in increasing succinate production.

Figure S3 summarizes how the various reported metabolic engineering techniques and the identified target metabolites help to enhance succinate production. This example demonstrates the utility of flux-sum analysis in identifying metabolite targets for metabolic engineering.


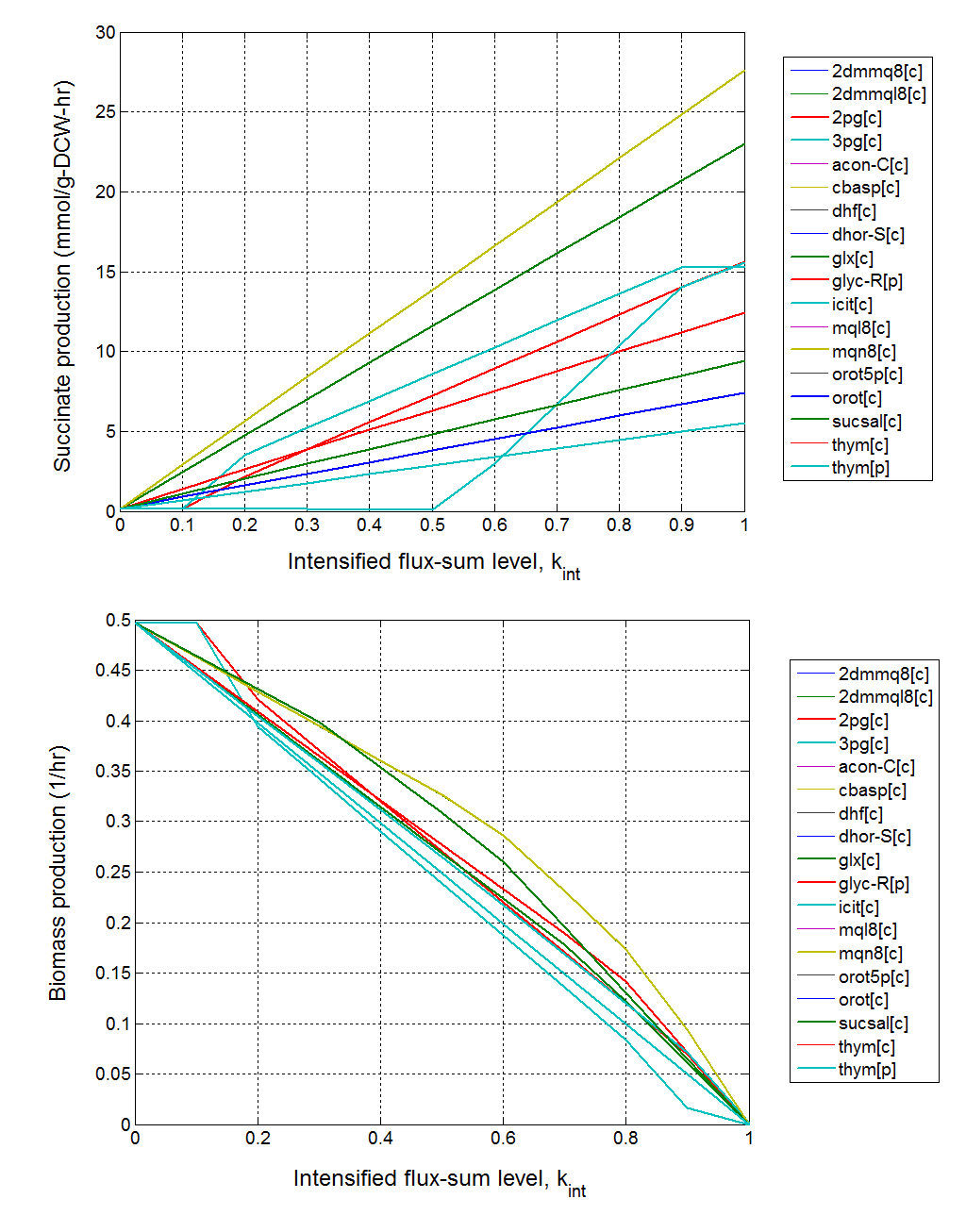


**Figure S2: Metabolite flux-sum intensification of potential targets.**

Abbreviations: 2dmmq8, 2-Demethylmenaquinone-8; 2dmmql8, 2-Demethylmenaquinol-8; 2pg, 2-phospho-D-glycerate; 3pg, 3-phosphoglycerate; acon-C, *cis*-aconitate; cbasp, N-carbamoyl-L-aspartate; dhf, 7,8-dihydrofolate; dhor-S, (S)-dihydroorotate; glx, glyoxylate; glyc-R, (R)-glycerate; icit, isocitrate; mql8, menaquinol-8; mqn8, menaquinone-8; orot5p, orotidine-5-phosphate; orot, orotate; sucsal, succinate semialdehyde; thym, thymine. [c] indicates cytosolic localisation and [p] indicates periplasmic localisation.


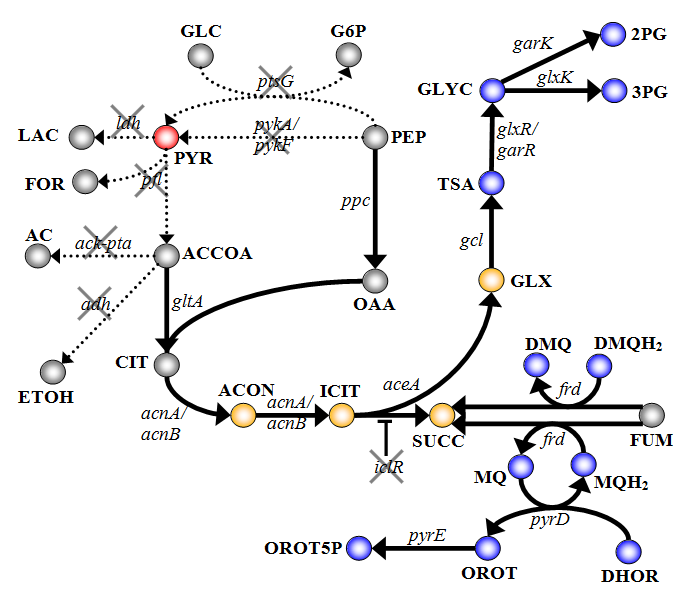


**Figure S3: Network map of identified targets.** Among the possible targets for flux-sum attenuation, the orange metabolites belong to the glyoxylate pathway and the blue metabolites belong to other various pathways. Pyruvate (red) is the only target for flux-sum attenuation. Other metabolites (grey) are not identified as potential targets in flux-sum analysis. Disruption of *iclR* removes the block on isocitrate lyase. Abbreviations: 2PG, 2-phospho-D-glycerate; 3PG, 3-phosphoglycerate; AC, acetate; ACCOA, acetyl-CoA; ACON, aconitate; CIT, citrate; DHOR, (S)-dihydroorotate; DMQ, demethylmenaquinone-8; DMQH_2_, demethylmenaquinol-8; ETOH, ethanol; FOR, formate; FUM, fumarate; G6P, glucose-6-phosphate; GLC, glucose; GLX, glyoxylate; GLYC, D-glycerate; ICIT, isocitrate; LAC, lactate; MQ, menaquinone-8; MQH_2_, menaquinol-8; OAA, oxaloacetate; OROT, orotate; OROT5P, orotidine-5-phosphate; PEP, phosphoenolpyruvate; PYR, pyruvate; SUCC, succinate; TSA, tartronate semialdehyde.

## Mathematical formulation for checking succinate production

$\min v_{EX\_succ}$ **(P4)**

s.t.

$$v_{biomass}\geq B_{k}$$

$$\sum_{j} S_{ij}v_{j}=0$$

$$S_{ij}v_{j}=f_{ij}^{+}-f_{ij}^{-}$$

**(C1):** $0.5\sum_{j} f_{ij}^{+}+f_{ij}^{-}\leq k_{\text{att}}\Phi_{i}^{B}$where $k_{\text{att}}\in\left[0,1\right]$

**OR**

**(C2):** $0.5\sum_{j} f_{ij}^{+}+f_{ij}^{-}\geq\Phi_{i}^{B}+k_{int}\left(\Phi_{i}^{u}-\Phi_{i}^{B}\right)$where $k{}_{\text{int}}\in\left[0,1\right]$

$$f_{ij}^{+}\leq I_{ij}^{+}\cdot M$$

$$f_{ij}^{-}\leq I_{ij}^{-}\cdot M$$

$I_{ij}^{+}+I_{ij}^{-}=1$.

$\alpha_{j}\leq v_{j}\leq\beta_{j}$for some$\alpha_{j},\beta_{j}$∈ and $\alpha_{j}\leq\beta_{j}$(Flux capacity constraints)

$\lambda_{i}\leq v_{EX\_i}\leq\mu_{i}$for some$\lambda_{i},\mu_{i}$∈ and $\lambda_{i}\leq\mu_{i}$(Uptake/secretion constraints)

$f_{ij}^{+}\geq0$, $f_{ij}^{-}\geq0$, $I_{ij}^{+}\in\{0,1\}$, $I_{ij}^{-}\in\{0,1\}$

In the above formulation, $B_{k}$ refers to the maximum allowable biomass production value at the given $k_{\text{att}}$ or $k_{\text{int}}$ value. The subscript *k* indicates that this value depends on the value of the flux-sum constants $k_{\text{att}}$ or $k_{\text{int}}$. The values of $B_{k}$ are solutions to of **(P3)** for every value of $k_{\text{att}}$ or $k_{\text{int}}$.

# References

Lee SJ, Lee D-Y, Kim TY, Kim BH, Lee J, Lee SY: **Metabolic Engineering of Escherichia coli for enhanced production of succinic acid, based on genome comparison and in silico gene knockout simulation**. *Applied and Environmental Microbiology* 2005, **71**(12), pp. 7880-7887.

Sanchez AM, Benett GN, San K-Y: **Novel pathway engineering design of the anaerobic central metabolic pathway in *Escherichia coli* to increase succinate yield and productivity**. *Metabolic Engineering* 2005, **7**, pp. 229-239.

Wang Q, Chen X, Yang Y, Zhao X: **Genome-scale *in silico* aided metabolic analysis and flux comparisons of Escherichia coli to improve succinate production**. *Appl Microbiol Biotechnol* 2006, **73**, pp. 887-894.
